# National School Health Survey: Methodological aspects changes and comparability with the Global School-based Student Health Survey

**DOI:** 10.1590/1980-549720240053

**Published:** 2024-11-22

**Authors:** Alan Cristian Marinho Ferreira, Alanna Gomes da Silva, Évelin Angélica Herculano de Morais, Deborah Carvalho Malta

**Affiliations:** I Universidade Federal de Minas Gerais, School of Nursing, Graduate Program in Nursing, Belo Horizonte (MG), Brazil.

**Keywords:** Adolescents, Public health surveillance, Health surveys, Health status indicators

## Abstract

**Objective:**

To analyze the changes in the methodological aspects of the National Survey of School Health (PeNSE) and its comparability with the Global School-based Student Health Survey (GSHS).

**Methods:**

This evaluative study that utilized the PeNSE questionnaires from 2009, 2012, 2015, and 2019, and the GSHS questionnaires from 2013-2017 e 2018-2020. The variables analyzed included the sample size, representativeness and geographic stratification of PeNSE, the number of questions in PeNSE, the percentage similarity of the PeNSE 2019 relative to the 2015, and its comparability with GSHS.

**Results:**

Over the four editions of PeNSE, the sample size increased (from 63,411 in 2009 to 125,123 in 2019). There were changes in educational levels (exclusion of the 6th grade and inclusion of the 7th and 8th grades of primary and secondary education), geographic stratification (expanded to large regions and federation units), and the number of questions increased by 46%. Regarding the similarity between the 2015 and 2019 editions, 48 questions were added, 35 were excluded, and 4 were changed. In 2019, PeNSE presented 11 modules that were partially comparable and 3 that were potentially not to those of 2015. The PeNSE 2015 edition was more similar to the GSHS, with 10 comparable modules, whereas in 2019, this number was reduced to five.

**Conclusion:**

Since its creation, PeNSE has undergone several changes, including increased sample representativeness and number of questions across editions. However, changes to the questionnaires must be analyzed with caution, as they may compromise comparability with previous editions and international surveys.

## INTRODUCTION

The National School Health Survey (PeNSE) is a study conducted by the Brazilian Ministry of Health in partnership with the Brazilian Institute of Geography and Statistics (*Instituto Brasileiro de Geografia e Estatística* – IBGE), initiated in 2009. Its objective is to gather data on the health and well-being of elementary and high school students from both public and private schools across the country^1^.

The survey was designed to be conducted every three years and is regarded as a key component of the health surveillance system for Brazilian adolescents. It allows for the monitoring of health risk and protective factors, particularly those associated with noncommunicable diseases (NCDs), such as diet, physical activity, and the use of legal and illegal substances^2^. Monitoring adolescent health through PeNSE is part of Brazil's efforts to fulfill global commitments to reduce NCDs by preventing their risk factors and providing appropriate care to affected individuals^3^.

To be truly effective in the health surveillance process and contribute to reducing NCDs risk factors, PeNSE must ensure consistency in its metrics, enabling comparability of results across its editions and with international surveys such as the Global School-based Student Health Survey (GSHS). This requires maintaining existing questions while adding new ones as necessary^2,4^. However, changes have occurred over the editions. The first edition of PeNSE focused on 9^th^-grade elementary school students from all state capitals and the Federal District. By the 2019 edition, the target population had expanded to include students from the 7^th^ grade of elementary school through the 3^rd^ year of high school, aged 13 to 17, from across the entire country. Additionally, there were modifications to the questionnaire, involving the inclusion and exclusion of questions, as well as adjustments to question formats and response scales.

These changes in both the sampling process and the data collection instruments over the years must be made with caution to avoid affecting comparability between editions, which could undermine the surveillance system, the monitoring of health indicators, and the interpretation and analysis of data. This highlights the importance of studies that evaluate these changes over time to contribute to future editions, minimize potential analytical inconsistencies when comparing variables across years, and ensure comparability between national and international research, as recommended by the World Health Organization (WHO).

In this context, the objective of this study was to assess the methodological changes in the National Survey of School Health (PeNSE) and its comparability with the Global School-based Student Health Survey.

## METHODS

### Study design

This is an evaluative study that utilized the PeNSE student questionnaires from the 2009, 2012, 2015, and 2019 editions, as well as the PeNSE results books published by IBGE, to conduct a comparative evaluation of sample size, representativeness, geographic stratification, and the number of questions. To assess similarity, the materials from the 2015 and 2019 editions were compared with the GSHS student questionnaires corresponding to the periods 2013–2017 and 2018–2020.

### PeNSE

PeNSE was the first Brazilian survey to examine the behavioral aspects of students in public and private schools across the country, while also addressing issues related to the family and school environment^1^. Utilizing a two-stage cluster sampling process (schools and classes), its sample comprised all students enrolled in the selected classes who were present at school on the day the survey was conducted and met the inclusion criteria^1^.

### Global School-based Student Health Survey

GSHS was created by the WHO in partnership with the United Nations Children's Fund (UNICEF), the United Nations Educational, Scientific and Cultural Organization (UNESCO), the Joint United Nations Programme on HIV/AIDS (UNAIDS), and the Centers for Disease Control and Prevention (CDC) in 2003, aiming to assess various health aspects of students aged 13 to 17 worldwide. Made available to over one hundred countries for use and adaptation to local contexts, it is one of the largest population surveys globally, particularly among Latin American countries^5^. GSHS employs a standardized sample selection process based on schools^4^ and collects data through a questionnaire that addresses a wide range of health-related topics, including mental health, physical activity, dietary habits, and the use of alcohol, tobacco, and other drugs, among others^4^. This tool helps identify the main health challenges faced by students worldwide and guides health promotion and disease prevention initiatives within the school population^5^. Additionally, it provides crucial information on health disparities between countries and can be used to assess the impact of public health interventions^5^.

The GSHS questionnaires are available on the World Health Organization website (https://www.who.int/teams/noncommunicable-diseases/surveillance/systems-tools/global-school-based-student-health-survey).

A summary of the main features of both surveys is provided in Supplementary Material I.

### Study variables

The following variables were analyzed in this study:

- sample used by PeNSE in each edition;
- representativeness of PeNSE in each edition;
- geographic stratification of PeNSE in each edition;
- number of questions in PeNSE, in each edition, by thematic module and total;
- percentage of similarity of the PeNSE 2019 questionnaire in relation to the PeNSE 2015 questionnaire;
- percentage of similarity of the PeNSE 2015 and 2019 questionnaires in relation to the GSHS 2013–2017 and 2018–2020 questionnaire;
- classification of the PeNSE question modules in relation to their comparability to the GSHS question modules.

Variables a and b were collected from the PeNSE results books, while variables d to g were extracted from the PeNSE and GSHS student questionnaires. For extraction, all questions from the questionnaires were tabulated in a Microsoft Office Excel^®^ spreadsheet, with columns organized by year of completion.

### Data analysis

Due to sampling considerations, only the results from the last two editions of PeNSE could be used in comparative analyses. This explains the focus on the similarity analysis for the 2015 and 2019 editions of the survey, as well as the comparability analysis with the GSHS.

To calculate the percentage of similarity between the 2015 and 2019 editions of PeNSE, the questions within each thematic module of the research were classified as follows:

- NC (no changes) — a question in 2019 that did not present any changes compared to the 2015 edition, therefore comparable;
- SQ (similar question) — a question in 2019 that was similar to the 2015 edition, still comparable;
- CQ (change in the question) — a question that underwent a change in the question in 2019 compared to 2015, becoming non-comparable;
- CR (change in the response) — a question that underwent a change in the response in 2019 compared to 2015, becoming non-comparable;
- EQ (excluded question) — a question that existed in the 2015 edition, but was deleted in the 2019 edition, making it non-comparable;
- NQ (new question) — a new question that was included in the questionnaire in 2019, which did not exist in the 2015 edition, thus non-comparable.

The percentage of similarity was calculated using the following formula:


$$\frac{\left(SA+QS\right)}{C}x100$$

Where C represents the total number of questions in the module in PeNSE 2015.

The questions were independently evaluated by peers, and in cases of disagreement, a third evaluator was consulted. The similarity analysis between PeNSE 2015 and 2019, in relation to the GSHS, followed the same methods, using the GSHS student questionnaire as a reference. According to the WHO, for a survey to be considered comparable to the GSHS, it must include at least six similar modules, with countries having the option to include additional questions^4^. In this research, a 60% similarity criterion between questions was adopted for analysis purposes.

### Ethical procedures

In all editions of PeNSE, students invited to participate consented through an Informed Consent presented at the beginning of the questionnaire, with the freedom to decide whether to participate. Although there were no direct risks to students’ health, the sensitivity of the topics addressed was considered, and measures were taken to protect their well-being and comfort. Participation was voluntary, allowing students to skip any question or choose not to complete the questionnaire. Their information remained confidential, and the schools were not identified.

All editions of PeNSE received approval from the National Commission for Research Ethics (*Comissão Nacional de Ética em Pesquisa –* CONEP) of the National Health Council (*Conselho Nacional de Saúde* – CNS), which oversees and approves health research involving human subjects. The CONEP approval numbers were: 11.537/2009 (2009 edition), 16.805/2012 (2012 edition), 1.006.467/2015 (2015 edition), and 3.249.268/2019 (2019 edition). Similarly, all countries conducting research through the GSHS obtain ethical approvals from their respective national government agencies and institutional ethics committees.

## RESULTS

Throughout its various editions, PeNSE has undergone sample modifications, as detailed in Chart 1. With each edition, the number of schools and students surveyed increased. The 2009 edition included 1,507 schools and a final valid sample of 63,411 students. In the most recent edition, the sample expanded to 4,361 schools with a final valid sample of 125,123 students (Chart 1).

**Chart 1:** Sampling aspects of the National School Health Survey in each edition. *Pesquisa Nacional de Saúde do Escolar*, 2009, 2012, 2015, and 2019.

Characteristics		2009	2012	2015 (sample 1)	2015 (sample 2)	2019
Planned sample						
	Schools	1,507	3,004	3,160	380	4,361
	Classes	2,270	4,288	4,418	652	6,803
	Enrolled students	72,596	131,741	128,027	19,558	187,957
Studied sample						
	Schools	1,453	2,842	3,040	371	4,242
	Classes	2,175	4,091	4,159	653	6,612
	Enrolled students	72,782	134,310	124,227	20,516	189,857
	Attending students	68,735	132,123	120,122	19,402	183,264
	Present students	63,411	110,873	120,122	16,608	183,264
	Responding students	63,411	109,104	102,301	16,556	160,721
	Representativeness	Students in the 9^th^ grade of ES; 26 Brazilian capitals; FD.	Students in the 9^th^ grade of ES; 26 Brazilian capitals; FD; Major Regions.	Students in the 9^th^ grade of ES; 26 Brazilian capitals; FD; FU, Major Regions; Brazil.	Students from the 6^th^ grade of ES to the 3^rd^ year of HS; ages 13 to 17; 26 Brazilian capitals; Major Regions; Brazil.	Students from the 7^th^ grade of ES to the 3^rd^ year of HS; ages 13 to 17; Brazil.
	Possible stratifications	Brazilian capitals and FD.	Brazilian capitals and FD, non-capitals, Major Regions.	Brazilian capitals and FD, non-capitals, states and FD, Major Regions.	Major Regions.	Major Regions, FU, municipalities of the capitals.

All editions included public and private schools. ES: elementary school. HS: high school. FD: Federal District. FU: Federative Units.

In terms of representativeness, the 2009 PeNSE survey was representative of public and private schools, 9^th^-grade elementary students, the 26 Brazilian capitals, and the Federal District. In 2012, representativeness was expanded to include Brazil's Major Regions, and in 2015 (sample 1), it extended to the entire country. The 2015 edition (sample 2) represented public and private schools, students from the 6th grade of elementary school to the 3^rd^ year of high school, students aged 13 to 17, the 26 Brazilian capitals, Major Regions, and Brazil. In the most recent edition, PeNSE was representative of public and private schools, students from the 7^th^ grade of elementary school to the 3^rd^ year of high school, students aged 13 to 17, and the entire country (Chart 1).

In terms of geographic stratification, the first edition of PeNSE allowed stratification only for Brazilian capitals and the Federal District. In the second edition, stratification was expanded to include the Major Regions. In the third edition, for sample 1, geographic stratification was possible for capitals, the Federal District, non-capitals, states, and Major Regions. Sample 2, however, was limited to stratification for Major Regions and Brazil. The latest edition enabled stratification for Major Regions, Federative Units, and municipalities within the capitals (Chart 1).

Table 1 presents the thematic modules for each edition of PeNSE, highlighting changes in the number of questions within each module and in the total number of questions in the student questionnaire. In the first edition, the survey contained 108 questions, while by 2019, this number increased to 158, representing a 46% growth (Table 1).

**Table 1:** Number of questions in the student questionnaire of the National School Health Survey, by thematic module and total. *Pesquisa Nacional de Saúde do Escolar*, 2009, 2012, 2015, and 2019.

Thematic module	Total			
	2009	2012	2015	2019
General information	26	20	25	20
Nutrition	19	20	17	26
Physical activity	13	11	12	10
Tobacco use	8	8	9	13
Alcohol consumption	11	9	8	9
Illicit drugs	0	5	6	6
Situations at home and school	4	9	10	10
Mental health	0	3	3	6
Sexual and reproductive health	9	10	12	13
Hygiene and oral health	4	6	6	6
Safety	10	13	18	24
Use of health services	0	4	8	9
Body image	2	5	7	6
Asthma	0	2	2	0
Weight and height	2	2	2	0
Total number of questions in the edition	108	128	145	158

Of the 15 thematic modules, PeNSE 2019 showed one module (Oral Hygiene and Health) with 100% similarity to the 2015 edition. Eleven modules had at least 60% similarity (general information 68%, diet 65%, physical activity 75%, cigarette use 89%, alcoholic beverages 88%, illicit drugs 83%, situations at home and at school 60%, sexual and reproductive health 83%, safety 72%, use of health services 88%, and body image 86%) (Table 2). Supplementary Material II provides a detailed comparison of all the questions from PeNSE 2015 and their counterparts in 2019, indicating whether the question remained unchanged, was similar, underwent changes in wording or response options, was excluded, or was newly added.

**Table 2:** Number of questions and percentage of similarity of thematic modules in the National School Health Survey from the 2019 edition compared to the 2015 edition.

Thematic module	IQ	SQ	UQ	Comparable questions between 2015 and 2019	% similarity in 2019
General information	16	1	2	18	68
Nutrition	10	1	15	11	65
Physical activity	9	0	1	9	75
Tobacco use	5	3	5	8	89
Alcohol consumption	6	1	2	7	88
Illicit drugs	4	1	1	5	83
Situations at home and school	6	0	4	6	60
Mental health	1	0	5	1	33
Sexual and reproductive health	7	3	3	10	83
Hygiene and oral health	2	4	0	6	100
Safety	9	4	11	13	72
Use of health services	7	0	2	7	88
Body image	6	0	0	6	86
Asthma	0	0	0	0	0
Weight and height	0	0	0	0	0

IQ: Identical questions; SQ: Similar questions; UQ: Unique questions; Unique questions: Questions that exist only in the 2019 edition = New Questions (NQ) + Questions with changes in the Question (QQ) + Questions with changes in the Answers (QR).

In terms of comparability between the PeNSE and GSHS surveys, the 2015 edition had the highest number of modules comparable to the international survey, with eight modules in total, seven of which were considered 100% comparable. By 2019, the modules "Dietary Behavior," "Hygiene," and "Violence and Unintentional Injury" were considered potentially non-comparable, reducing the number of comparable modules to six, with only two deemed 100% comparable (Table 3).

**Table 3:** Comparability between the National School Health Survey and the Global School-based Student Health Survey. PeNSE, 2015 and 2019, Global School-Based Student Health Survey, 2015–2018.

Thematic module	PeNSE 2015		PeNSE 2019	
	% of similarity	Classification in relation to the GSHS	% of similarity	Classification in relation to the GSHS
Demographic	100	Comparable	100	Comparable
Alcohol consumption	100	Comparable	100	Comparable
Dietary behavior	100	Comparable	57	Potentially non-comparable
Drug use	50	Potentially non-comparable	50	Potentially non-comparable
Hygiene	100	Potentially non-comparable	25	Potentially non-comparable
Mental health	50	Potentially non-comparable	17	Potentially non-comparable
Physical activity	100	Comparable	75	Comparable
Protective factors	100	Comparable	67	Comparable
Sexual behaviors contributing to HIV infection, other STIs, and unintended pregnancy	100	Comparable	80	Comparable
Tobacco use	83	Comparable	67	Comparable
Violence and unintentional injury	86	Comparable	25	Potentially non-comparable
HIV and Aids	0	Potentially non-comparable	0	Potentially non-comparable

GSHS: Global School-Based Student Health Survey; HIV: human immunodeficiency virus; STI: sexually transmitted infections.

Supplementary Material III provides the complete set of questions from the GSHS student questionnaire and a comparison with the 2015 and 2019 PeNSE editions.

## DISCUSSION

Throughout its editions, PeNSE underwent changes in its sample size, representativeness, and geographic stratification, expanding the number of participating schools and students, educational levels, age range, and possibilities for geographic stratification. Additionally, the total number of questions increased, along with adjustments in the number of questions within each thematic module. In terms of similarity between the 2015 and 2019 editions, most modules were partially comparable, with only one being fully comparable. The modules related to mental health, asthma, and anthropometric data were deemed potentially non-comparable. Regarding comparability with the GSHS, the 2015 edition of PeNSE had more comparable modules than the 2019 edition.

Since its inception, PeNSE has employed a random probabilistic sampling technique, based on clusters, using data from the most recent school census available for the year in which the survey was conducted^1^. This method is a strength of the survey's design, as it is compatible with the statistical analyses commonly used in quantitative epidemiological studies (confidence interval, hypothesis testing, regressions, etc.)^6^. Due to the probabilistic sampling, PeNSE is representative of schoolchildren across the entire country, granting studies based on PeNSE data a high level of inference power for this population^7^.

It is important to emphasize that analyses of PeNSE data must adhere to the sampling plan, including weights and similar calculations, to adjust for potential selection biases, such as the cluster effect. Furthermore, changes in sampling over the years, particularly regarding the target population, limit comparisons to students enrolled in the 9^th^ grade of elementary school, typically aged 13 to 15 years old, as this group remains consistent across all survey editions.

Regarding the representativeness and geographic stratification of the data, the agencies responsible for PeNSE consistently strive to enhance the detail of the data collected by the survey. This effort is crucial, as Brazil's continental dimensions can lead to significant variations in the scenarios investigated across different regions of the country^8^. A representative survey with stratification for Major Regions and states enables a better understanding of health determinants at local levels, assisting in the identification of priorities that support more specific and effective actions for health protection and promotion^9,10^.

The total number of questions in PeNSE has significantly expanded across its editions, enhancing the survey's investigative power by allowing the exploration of various aspects of individuals’ lives^11^. Furthermore, new questions can be incorporated to address contemporary issues, such as the inclusion of questions related to cyberbullying in the 2019 edition.

Regarding the comparability of PeNSE between its editions, the results of this study indicated weaknesses stemming from the incomparability of thematic modules. This situation contradicts the recommendations from organizations such as the European Centre for Disease Prevention and Control (ECDC) and CDC, which define quality attributes that all public health surveillance systems should encompass. These organizations emphasize the importance of consolidating systems that facilitate comparative analyses, whether within the same system over time for examining temporal trends or between external systems, such as those in other countries^12,13^.

Due to changes in the questionnaires between editions, such as deletions, additions of new questions, and modifications to response options, researchers must carefully examine the questionnaire before attempting to compare the surveys. For instance, the response options for bullying were altered in 2019, rendering them incomparable with previous editions^14^. Similarly, the removal of the question about adolescent work in 2019 hinders the ability to track this significant issue over time.

In comparison with international surveys like the GSHS, PeNSE showed a decrease in comparability in its most recent 2019 edition, which may hinder cross-country comparisons despite meeting the minimum number of comparable modules. Such changes could present obstacles to the promotion and protection of adolescent health in Brazil, as conducting an internationally comparable survey enables the evaluation of national epidemiological patterns relative to other countries. This also fosters dialogue between nations and the creation of collaboration networks that support health management and the proposal of more effective public health measures^15,16^.

In 2015, a significant effort was made to align PeNSE with the GSHS, resulting in eight similar modules; however, by 2019, changes in several questions reduced this similarity to six modules. Important global monitoring indicators, such as those related to physical activity and hunger, were removed in 2019, further hindering comparability^1,4^. As a result, Brazil has not yet succeeded in joining the WHO list of over one hundred countries that have conducted health surveys on their adolescent populations, due to these ongoing challenges in aligning PeNSE with GSHS^4^.

The limitations of this study include the procedures for comparability. Due to changes in sampling, it was not possible to extend the analysis to earlier editions of PeNSE (2009 and 2012). The choice of the 60% cutoff point for the similarity analyses was a decision made by the researchers.

A key strength of this study is the recognition of PeNSE as the primary tool for monitoring the health of Brazilian schoolchildren. The current analysis seeks to assist policymakers in achieving international comparability by identifying similarities between PeNSE and GSHS. Furthermore, the comprehensive review of the questionnaires enables the identification of significant changes over time, supporting future analyses.

Over the years, PeNSE has expanded in several aspects, including the number of participating schools and students, education levels, age range, geographic data stratification options, and the total number of questions. Between the 2015 and 2019 editions, most modules are partially comparable, with only one module being fully comparable. As for comparability with the GSHS, only the 2015 edition was deemed comparable.

PeNSE has undergone various changes, both in methodological aspects such as sampling, representativeness, and geographic stratification, and in the content of the student questionnaires. These changes risk undermining the stability of the survey and compromise its comparability, both across its own editions and with international surveys like the GSHS. Such alterations may hinder critical efforts, such as the analysis of temporal trends in health indicators among Brazilian adolescents. Strengthening the comparability of the Brazilian survey with international surveys is recommended, and to achieve this, the PeNSE student questionnaire should align as closely as possible with the GSHS.

PeNSE is widely recognized for its representativeness and its established significance in national scientific research. However, researchers must exercise caution when using its data. Analyses that involve different editions of the survey or international comparisons through the GSHS should be carefully scrutinized to ensure consistency in how the information is collected, as well as in the representativeness of the target population being studied.
