# Supplementary material for: National School Health Survey: Methodological aspects changes and comparability with the Global School-based Student Health Survey
Source: Rev Bras Epidemiol. 2024 Nov 22;27:e240053. doi: 10.1590/1980-549720240053 (PMC11654285; doi:10.1590/1980-549720240053)
Supplement: Translation

# Translation: Pesquisa Nacional de Saúde do Escolar: mudanças metodológicas e comparabilidade com o Global School-based Student Health Survey

**DOI:** 10.1590/1980-549720240053.2

**Authors:** Alan Cristian Marinho Ferreira, Alanna Gomes da Silva, Évelin Angélica Herculano de Morais, Deborah Carvalho Malta

**Affiliations:** I Universidade Federal de Minas Gerais, Escola de Enfermagem, Programa de Pós-Graduação em Enfermagem, Belo Horizonte (MG), Brasil.

**Keywords:** Adolescentes, Vigilância em saúde, Inquéritos epidemiológicos, Indicador de saúde

## Abstract

**Objetivo:**

Analisar as mudanças ocorridas nos aspectos metodológicos da Pesquisa Nacional de Saúde do Escolar (PeNSE) e sua comparabilidade com o *Global School-Based Student Health Survey* (GSHS).

**Métodos:**

Estudo avaliativo que utilizou o questionário da PeNSE de 2009, 2012, 2015 e 2019 e o do GSHS de 2013–2017 e 2018–2020. Analisaram-se as variáveis: amostra utilizada; representatividade e estratificação geográfica da PeNSE; número de questões da PeNSE; percentual de similaridade da PeNSE 2019 em relação à de 2015 e à sua comparabilidade ao GSHS.

**Resultados:**

Ao longo das quatro edições, a amostra aumentou (de 63.411 em 2009 para 125.123 em 2019), houve mudanças nos graus de escolaridade (exclusão do 6° ano e inclusão do 7° e 8° anos do ensino fundamental e ensino médio), na estratificação geográfica (ampliou para Grandes Regiões, unidades de federação), e o número de questões aumentou 46%. Quanto à similaridade entre as edições de 2015 e 2019, 48 questões foram inseridas, 35 excluídas e quatro foram alteradas. Em 2019, a PeNSE apresentou 11 módulos parcialmente e três pontencialmente não comparáveis à de 2015. A edição da PeNSE 2015 foi mais similar ao GSHS, contando com oito módulos comparáveis e, em 2019, esse número reduziu para seis.

**Conclusão:**

Desde sua criação, a PeNSE passou por diversas mudanças: houve aumento de representatividade da amostra e número de questões ao longo das edições. Entretanto, as mudanças nos questionários devem ser analisadas com cautela, uma vez que podem comprometer a comparabilidade com edições anteriores e com inquéritos internacionais.

## INTRODUÇÃO

A Pesquisa Nacional de Saúde do Escolar (PeNSE) é um inquérito do Ministério da Saúde do Brasil, em parceria com o Instituto Brasileiro de Geografia e Estatística (IBGE), iniciado em 2009. O objetivo da pesquisa é coletar informações sobre a saúde e o bem-estar de estudantes do ensino fundamental e médio de escolas públicas e privadas do país^1^.

A pesquisa foi planejada para ser realizada trienalmente, sendo considerada como o principal componente do sistema de vigilância em saúde dos adolescentes brasileiros, pois possibilita o acompanhamento dos fatores de risco e proteção da saúde, sobretudo os relacionados às doenças crônicas não transmissíveis (DCNT), como alimentação, atividade física e o uso de drogas lícitas e ilícitas^2^. O monitoramento da saúde dos adolescentes, operacionalizado por meio da PeNSE, integra os esforços brasileiros para a efetivação, em território nacional, dos compromissos globais de redução das DCNT por meio da prevenção de seus fatores de risco, bem como a oferta de atenção adequada aos doentes^3^.

Para que seja de fato efetiva no processo de vigilância em saúde e auxilie na redução dos fatores de risco para as DCNT, a PeNSE deve garantir a consistência de suas métricas, permitindo a comparabilidade de seus resultados entre suas edições e com inquéritos internacionais como o *Global School-based Student Health Survey* (GSHS), mantendo perguntas já existentes e acrescentando novas, caso necessário^2,4^. Contudo, mudanças ocorreram ao longo das edições. A primeira edição da PeNSE tinha como população alvo alunos do 9° ano do ensino fundamental, de todas as capitais e do Distrito Federal. Já na edição de 2019, a população alvo abrangeu alunos do 7° ano do ensino fundamental ao 3° ano do ensino médio, de 13 a 17 anos, de todo o território nacional. Além disso, houve mudanças no questionário, envolvendo inclusão e exclusão de questões, ajustes no formato das perguntas e nas escalas das respostas.

Essas alterações tanto no processo amostral quanto no instrumento de coleta de dados ao longo das edições devem ser realizadas com cautela a fim de não impactar a comparabilidade entre os anos, comprometendo o sistema de vigilância, o monitoramento dos indicadores de saúde, as interpretações e análises dos dados. Assim, reforça-se a importância de estudos que avaliem as mudanças ao longo dos anos, a fim de contribuir para as futuras edições, minimizar possíveis inconsistências analíticas ao comparar as variáveis entre os anos e possibilitar a comparação da pesquisa nacional com as internacionais como é preconizado pela Organização Mundial da Saúde (OMS).

Nesse sentido, o objetivo deste estudo foi avaliar mudanças metodológicas da Pesquisa Nacional de Saúde do Escolar e a sua comparabilidade com o *Global School-based Student Health Survey*.

## MÉTODOS

### Delineamento do estudo

Trata-se de um estudo avaliativo que utilizou os questionários do aluno da PeNSE nas edições 2009, 2012, 2015 e 2019 e os livros de resultados da PeNSE divulgados pelo IBGE para a avaliação comparativa do tamanho da amostra, representatividade, estratificação geográfica e número de questões. Para a avaliação da similaridade, foram utilizados os materiais das edições 2015 e 2019, os questionários do aluno do GSHS equivalentes ao período de 2013–2017 e 2018–2020.

### A PeNSE

A PeNSE foi o primeiro inquérito brasileiro que investigou em âmbito nacional aspectos comportamentais dos escolares da rede pública e privada de ensino, além de abordar questões quanto ao ambiente familiar e escolar^1^. Utilizando um processo de amostragem por conglomerado em dois estágios (escolas e turmas), tem sua amostra composta de todos os alunos matriculados das turmas selecionadas, presentes no dia da realização da pesquisa na escola, e que atendiam aos critérios de inclusão^1^.

### O Global School-based Student Health Survey

O GSHS é um inquérito criado pela OMS em parceria com o Fundo das Nações Unidas para a Infância (UNICEF), a Organização das Nações Unidas para a Educação, a Ciência e a Cultura (UNESCO), o Programa Conjunto das Nações Unidas sobre HIV/AIDS (UNAIDS) e os Centers for Disease Control and Prevention (CDC) em 2003, com o intuito de avaliar aspectos relacionados à saúde de estudantes de 13 a 17 anos em todo o mundo. Disponibilizado a mais de cem países para uso e adaptação conforme o contexto local, trata-se de uma das maiores pesquisas populacionais do mundo, principalmente entre os países latino-americanos^5^. Também utiliza um processo de seleção de amostra padronizado baseado nas escolas^4^, realiza a coleta de dados por meio de um questionário que abrange uma variedade de temas relacionados à saúde, incluindo saúde mental, atividade física, hábitos alimentares, uso de álcool, tabaco e outras drogas, entre outros^4^. É uma ferramenta que ajuda a identificar os principais problemas de saúde enfrentados pelos estudantes em todo o mundo e orientar as ações de promoção da saúde e prevenção de doenças na população escolar^5^. Além disso, fornece informações importantes sobre as diferenças na saúde entre os países e pode ser usado como um instrumento para avaliar o impacto de intervenções de saúde pública^5^.

Os questionários referentes ao GSHS estão disponíveis no *site* da Organização Mundial da Saúde (https://www.who.int/teams/noncommunicable-diseases/surveillance/systems-tools/global-school-based-student-health-survey).

Uma síntese das principais características de ambos os inquéritos estão disponíveis no Material Suplementar I.

### Variáveis do estudo

Analisaram-se neste estudo as seguintes variáveis:

- amostra utilizada pela PeNSE em cada edição;
- representatividade da PeNSE em cada edição;
- estratificação geográfica da PeNSE em cada edição;
- número de questões existentes na PeNSE, em cada edição, por módulo temático e total;
- percentual de similaridade do questionário da PeNSE 2019 em relação ao questionário da PeNSE 2015;
- percentual de similaridade dos questionários da PeNSE 2015 e 2019 em relação ao questionário do GSHS 2013–2017 e 2018–2020;
- classificação dos módulos de questões da PeNSE em relação à sua comparabilidade aos módulos de questões do GSHS.

As variáveis a e b foram coletadas dos livros de resultados da PeNSE. Já as variáveis de d a g foram extraídas do questionário do aluno da PeNSE e do GSHS. Para esta extração, tabularam-se em uma planilha do programa Microsoft Office Excel^®^ todas as questões existentes nos questionários, divididas em colunas por ano de realização.

### Análises dos dados

Em razão dos aspectos amostrais, apenas os resultados das duas últimas edições da PeNSE poderiam ser utilizados em análises comparativas, o que justifica a análise de similaridade apenas paras as edições de 2015 e 2019 da pesquisa e também da análise de comparabilidade com o GSHS.

Para o cálculo do percentual de similaridade entre as edições 2015 e 2019 da PeNSE, classificaram-se as questões existentes em cada módulo temático da pesquisa da seguinte maneira:

- SA (sem alteração) — questão em 2019 que não apresentou alterações em relação à edição de 2015, portanto comparável;
- QS (questão similar) — questão em 2019 que era similar à edição de 2015, ainda comparável;
- AP (alteração na pergunta) — questão que sofreu alteração na pergunta em 2019 em relação a 2015, tornando-se incomparável;
- AR (alteração na resposta) — questão que sofreu alteração na resposta em 2019 em relação a 2015, tornando-se incomparável;
- QE (questão excluída) — questão existente na edição de 2015, mas excluída na edição de 2019, sendo incomparável;
- QN (questão nova) — questão nova que foi incluída no questionário em 2019, não existindo na edição de 2015, assim incomparável.

O cálculo do percentual de similaridade foi realizado utilizando a seguinte fórmula:


$$\frac{\left(SA+QS\right)}{C}\;x\;100$$

Em que C é igual ao número total de questões existente no módulo na PeNSE 2015.

A classificação das questões foi realizada por pares, de forma independente, e, em caso de divergência, um terceiro avaliador foi consultado. A análise de similaridade entre a PeNSE 2015 e 2019 em relação ao GSHS seguiu os métodos descritos acima, tendo como referência o questionário do aluno do GSHS. Para serem considerados comparáveis ao GSHS, a OMS estipulou pelo menos seis módulos semelhantes, podendo ser acrescentadas outras questões pelos países^4^. Para fins de análises desta pesquisa, adotou-se o critério de similaridade entre as perguntas de 60%.

### Procedimentos éticos

Para todas as edições da PeNSE, os alunos convidados que participaram da pesquisa consentiram por meio do Termo de Consentimento Livre e Esclarecido (TCLE) apresentado no início do questionário, tendo a liberdade de optar por responder ou não. Embora não tenha havido riscos diretos à saúde dos estudantes, foi considerada a sensibilidade das questões investigadas, adotando-se medidas para proteger seu bem-estar e conforto. A participação foi voluntária, permitindo aos alunos a escolha de não responder a qualquer pergunta ou o questionário completo, e suas informações foram mantidas em sigilo, sem identificação da escola.

Todas as edições da PeNSE foram aprovadas pela Comissão Nacional de Ética em Pesquisa (CONEP), do Conselho Nacional de Saúde (CNS), que regulamenta e aprova pesquisas em saúde envolvendo seres humanos. Os pareceres CONEP foram: 11.537/2009 (edição 2009), 16.805/2012 (edição 2012), 1.006.467/2015 (edição 2015) e 3.249.268/2019 (edição 2019). Todos os países que realizam a pesquisa por meio do GSHS obtêm aprovações éticas de suas respectivas agências governamentais nacionais e de comitê institucional de ética.

## RESULTADOS

A PeNSE apresentou, ao longo de suas edições diversas, modificações amostrais apresentadas no Quadro 1. Quanto à amostra utilizada, houve ampliação do número de escolas e escolares pesquisados em cada edição, tendo a edição de 2009 sido composta de 1.507 escolas e uma amostra final válida de 63.411 escolares. Na última edição da pesquisa, a amostra contou com 4.361 escolas e uma amostra final válida de 125.123 escolares (Quadro 1).

**Quadro 1:** Aspectos amostrais da Pesquisa Nacional de Saúde do Escolar em cada edição. Pesquisa Nacional de Saúde do Escolar, 2009, 2012, 2015 e 2019.

Características		2009	2012	2015 (amostra 1)	2015 (amostra 2)	2019
Amostra planejada						
	Escolas	1.507	3.004	3.160	380	4.361
	Turmas	2.270	4.288	4.418	652	6.803
	Matriculados	72.596	131.741	128.027	19.558	187.957
Amostra pesquisada						
	Escolas	1.453	2.842	3.040	371	4.242
	Turmas	2.175	4.091	4.159	653	6.612
	Matriculados	72.782	134.310	124.227	20.516	189.857
	Escolares frequentes	68.735	132.123	120.122	19.402	183.264
	Escolares presentes	63.411	110.873	120.122	16.608	183.264
	Escolares respondentes	63.411	109.104	102.301	16.556	160.721
	Representatividade	Alunos do 9° ano do EF; 26 capitais brasileiras; DF.	Alunos do 9° ano do EF; 26 capitais brasileiras; DF; Grandes Regiões.	Alunos do 9° ano do EF; 26 capitais brasileiras; DF; UF, Grandes Regiões; Brasil.	Alunos do 6° ano do EF ao 3° ano do EM; de 13 a 17 anos; 26 capitais brasileiras; Grandes Regiões; Brasil.	Alunos do 7° ano do EF ao 3° ano do EM; alunos de 13 a 17 anos; Brasil.
	Estratificações possíveis	Capitais brasileiras e DF.	Capitais brasileiras e DF, não capitais, Grandes Regiões.	Capitais brasileiras e DF, não capitais, estados e DF, Grandes Regiões.	Grandes regiões.	Grandes regiões, UF, municípios das capitais.

Todas as edições abrangeram escolas públicas e privadas. EF: ensino fundamental. EM: ensino médio. DF: Distrito Federal. UF: Unidades Federativas.

No que se refere à representatividade, em 2009 a PeNSE foi representativa para escolas públicas e privadas, alunos do 9° ano do ensino fundamental, 26 capitais brasileiras e para o Distrito Federal; e expandiu a representatividade para Grandes Regiões em 2012 e para o Brasil em 2015 amostra 1. Já na edição de 2015, amostra 2, a representatividade foi para escolas públicas e privadas, alunos do 6° ano do ensino fundamental ao 3° ano do ensino médio, alunos de 13 a 17 anos, 26 capitais brasileiras, Grandes Regiões e Brasil. A última edição da PeNSE foi representativa para escolas públicas e privadas, alunos do 7° ano do ensino fundamental ao 3° ano do ensino médio, alunos de 13 a 17 anos e Brasil (Quadro 1).

Quanto à estratificação geográfica possível dos dados, na primeira edição era possível a estratificação apenas para as capitais brasileiras e o Distrito Federal. Na segunda edição houve uma ampliação agregando as Grandes Regiões. Na terceira edição, na amostra 1, era possível a estratificação geográfica para capitais, Distrito Federal, não capitais, estados e Distrito Federal e Grandes Regiões. A amostra 2 contemplava apenas Grandes Regiões e Brasil. A última edição permitiu a estratificação para Grandes Regiões, Unidades da Federação e municípios das capitais (Quadro 1).

A Tabela 1 apresenta os módulos temáticos de cada edição da PeNSE. Percebe-se alteração no número de questões em cada módulo temático e no número total de questões existentes no questionário do aluno. Na primeira edição a pesquisa possuía 108 questões compondo seu instrumento de coleta de dados, e em 2019 esse instrumento contou com 158 questões (aumento de 46%) (Tabela 1).

**Tabela 1:** Número de questões existentes no questionário do aluno da Pesquisa Nacional de Saúde do Escolar, por módulo temático e total. Pesquisa Nacional de Saúde do Escolar, 2009, 2012, 2015 e 2019.

Módulo temático	Quantidade total			
	2009	2012	2015	2019
Informações gerais	26	20	25	20
Alimentação	19	20	17	26
Atividade física	13	11	12	10
Uso de cigarro	8	8	9	13
Bebidas alcoólicas	11	9	8	9
Drogas ilícitas	0	5	6	6
Situações em casa e na escola	4	9	10	10
Saúde mental	0	3	3	6
Saúde sexual e reprodutiva	9	10	12	13
Higiene e saúde bucal	4	6	6	6
Segurança	10	13	18	24
Uso dos serviços de saúde	0	4	8	9
Imagem corporal	2	5	7	6
Asma	0	2	2	0
Peso e altura	2	2	2	0
Total de perguntas na edição	108	128	145	158

Dos 15 módulos temáticos, com relação à edição de 2015, a PeNSE 2019 apresentou um módulo (Higiene e Saúde Bucal) com 100% de similaridade, 11 módulos com pelo menos 60% de similaridade (informações gerais 68%, alimentação 65%, atividade física 75%, uso de cigarro 89%, bebidas alcoólicas 88%, drogas ilícitas 83%, situações em casa e na escola 60%, saúde sexual e reprodutiva 83%, segurança 72%, uso de serviços de saúde 88% e imagem corporal 86%) (Tabela 2). No Material Suplementar II estão apresentadas de forma completa todas as perguntas contidas na PeNSE de 2015 e sua comparação com 2019, considerando se a questão não foi alterada, se foi similar, se houve alteração na pergunta ou resposta, se foi excluída ou nova.

**Tabela 2:** Número de questões e percentual de similaridade dos módulos temáticos da Pesquisa Nacional de Saúde do Escolar da edição 2019 em relação à de 2015.

Módulo temático	QI	QS	QU	Questões comparáveis entre 2015 e 2019	% similaridade em 2019
Informações gerais	16	1	2	18	68
Alimentação	10	1	15	11	65
Atividade física	9	0	1	9	75
Uso de cigarro	5	3	5	8	89
Bebidas alcoólicas	6	1	2	7	88
Drogas ilícitas	4	1	1	5	83
Situações em casa e na escola	6	0	4	6	60
Saúde mental	1	0	5	1	33
Saúde sexual e reprodutiva	7	3	3	10	83
Higiene e saúde bucal	2	4	0	6	100
Segurança	9	4	11	13	72
Uso de serviços de saúde	7	0	2	7	88
Imagem corporal	6	0	0	6	86
Asma	0	0	0	0	0
Peso e altura	0	0	0	0	0

QI: Questões idênticas; QS: Questões similares; QU: Questões únicas; Questões únicas: Questões existentes somente na edição 2019 = Questões Novas (QN) + Questões com alteração na Pergunta (QP) + Questões com alterações nas Respostas (QR).

Quanto à comparabilidade entre os inquéritos PeNSE e GSHS, a edição de 2015 apresentou o maior número de módulos comparáveis à pesquisa internacional, oito módulos no total, sendo sete considerados 100% comparáveis. Em 2019 os módulos "Comportamento Dietético", "Higiene" e "Violência e Lesão Não Intencional" foram considerados potencialmente incomparáveis, reduzindo para seis o número de módulos comparáveis ao questionário internacional, sendo dois considerados 100% comparáveis (Tabela 3).

**Tabela 3:** Comparabilidade entre a Pesquisa Nacional de Saúde do Escolar e o *Global School-based Student Health Survey*. PeNSE, 2015 e 2019, *Global School-Based Student Health Survey*, 2015–2018.

Módulo temático	PeNSE 2015		PeNSE 2019	
	% de similaridade	Classificação em relação ao GSHS	% de similaridade	Classificação em relação ao GSHS
Demográfico	100	Comparável	100	Comparável
Uso de álcool	100	Comparável	100	Comparável
Comportamento dietético	100	Comparável	57	Potencialmente incomparável
Uso de drogas	50	Potencialmente incomparável	50	Potencialmente incomparável
Higiene	100	Potencialmente incomparável	25	Potencialmente incomparável
Saúde mental	50	Potencialmente incomparável	17	Potencialmente incomparável
Atividade física	100	Comparável	75	Comparável
Fatores protetivos	100	Comparável	67	Comparável
Comportamentos sexuais que contribuem para infecção por HIV, outras IST e gravidez não planejada	100	Comparável	80	Comparável
Uso de tabaco	83	Comparável	67	Comparável
Violência e lesão não intencional	86	Comparável	25	Potencialmente incomparável
HIV e Aids	0	Potencialmente incomparável	0	Potencialmente incomparável

GSHS: *Global School-Based Student Health Survey*; HIV: vírus da imunodeficiência humana; IST: infecções sexualmente transmissíveis.

O Material Suplementar III apresenta as questões completas no questionário do aluno do GSHS e a comparação com a PeNSE de 2015 e 2019.

## DISCUSSÃO

Durante suas edições, a PeNSE apresentou mudanças em sua amostra, representatividade e estratificação geográfica dos dados, aumentando o número de escolas e escolares participantes, graus de escolaridade, faixa etária e possibilidades de estratificação geográfica dos dados. Houve ainda aumento do número total de questões, além de alterações no número de questões existentes em cada módulo temático. Quanto à similaridade entre as edições de 2015 e 2019, a maioria dos módulos é parcialmente comparável entre si, sendo apenas um totalmente comparável. Os módulos referentes à saúde mental, asma e dados antropométricos foram considerados potencialmente não comparáveis. Em relação à comparabilidade ao GSHS, a edição de 2015 da PeNSE apresentou mais módulos comparáveis, em relação à edição de 2019.

No que se refere à técnica de amostragem, desde a sua criação, a PeNSE utilizou um processo de amostragem probabilística aleatória, por conglomerados, baseada nas informações disponíveis no censo escolar mais recente em relação ao ano de realização da pesquisa^1^. Essa técnica representa uma fortaleza dos métodos utilizados pela pesquisa visto que a técnica é compatível com as análises estatísticas mais utilizadas nos estudos epidemiológicos quantitativos (intervalo de confiança, testes de hipóteses, regressões etc.)^6^. Em função da amostragem probabilística, a PeNSE possui representatividade para os escolares de todo o território nacional; assim, os estudos produzidos utilizando os resultados da PeNSE possuem alto poder de inferência para essa população^7^.

Cabe ressaltar que as análises desses dados devem respeitar o plano amostral, pesos e similares calculadas, para que possíveis vieses de seleção sejam ajustados, como o efeito de conglomeração, neste caso. Além disso, as mudanças na amostragem ao longo das edições, no que tange ao público-alvo, permite apenas a comparação entre escolares matriculados no 9° ano do ensino fundamental, que geralmente abrange adolescentes de 13 a 15 anos, uma vez que representa o grupo comum para todos os anos.

Com relação à representatividade e estratificação geográfica dos dados, nota-se um esforço constante dos órgãos responsáveis pela PeNSE para potencializar o detalhamento possível dos dados obtidos pela pesquisa. Esse movimento é importante pois, tendo o Brasil dimensões continentais, podem-se apresentar variações nos cenários investigados em cada região do país^8^. Ter uma pesquisa representativa e com estratificação para Grandes Regiões e unidades federativas permite conhecer os determinantes de saúde da população em níveis locais, ajudando na identificação de prioridades que favoreçam ações mais específicas e efetivas de proteção e promoção da saúde^9,10^.

Acerca do número total de questões da PeNSE ao longo de suas edições, foi notável a expansão do número total de perguntas, o que pode ampliar o poder investigativo do inquérito, uma vez que permite a abordagem de diversos aspectos da vida do indivíduo^11^. Além disso, podem-se incluir novas questões de acordo com a necessidade da época, como é o caso do acréscimo, na edição 2019, da investigação referente ao *cyberbullying*, tema contemporâneo.

Quanto à comparabilidade da PeNSE entre suas edições, os resultados deste estudo mostraram que a pesquisa apresenta fragilidades por conta da incomparabilidade de módulos temáticos, o que vai contra as recomendações de órgãos como o European Centre for Disease Prevention and Control (ECDC) e os CDC. Eles definem atributos de qualidade que deveriam ser contemplados por todos os sistemas de vigilância em saúde pública e ressaltam a importância de consolidação de sistemas que permitam a realização de análises comparativas de seus componentes, seja entre o mesmo sistema, em diferentes momentos do tempo para análises de tendências temporais, seja entre sistemas externos, como os existentes em outros países^12,13^.

Em consequência das mudanças nos questionários entre as edições, com exclusões, introdução de novas questões, alterações nas opções de respostas, os pesquisadores precisam analisar em detalhes o questionário antes de proceder à comparação das pesquisas. Por exemplo, as opções de resposta de sofrer *bullying* foram modificadas em 2019, não podendo ser comparadas com as edições anteriores^14^. Outro exemplo é a supressão da pergunta sobre o trabalho entre adolescentes em 2019, impedindo o monitoramento dessa importante condição.

A respeito da comparação com inquéritos internacionais como o GSHS, a PeNSE apresentou diminuição de comparabilidade em sua última edição de 2019, o que pode prejudicar a comparação com outros países, apesar de ter apresentado o número mínimo de módulos comparáveis. Tais mudanças podem se tornar barreiras para a promoção e proteção da saúde dos adolescentes brasileiros, visto que a execução de um inquérito comparável internacionalmente permite a avaliação dos padrões epidemiológicos nacionais perante os encontrados em outros países e o diálogo entre nações, bem como a formação de redes de colaboração que apoiarão a gestão das ações em saúde e a proposição de medidas mais eficazes no contexto da saúde^15,16^.

Foi realizado um grande esforço de aproximação da PeNSE com o inquérito GSHS em 2015, que resultou em oito módulos similares; entretanto, em 2019, com as mudanças em várias questões, a similaridade foi encontrada para seis módulos. Indicadores de monitoramento global, como os referentes à prática de atividade física, fome e outros, foram retirados em 2019, impedindo a comparabilidade^1,4^. Dadas as dificuldades na comparabilidade entre PeNSE e GSHS, o Brasil ainda não conseguiu entrar para a lista da OMS que agrega os mais de cem países que já realizaram pesquisas de saúde em sua população adolescente^4^.

As limitações do estudo envolvem os procedimentos para comparabilidade, pois, diante das mudanças na amostragem, não foi possível ampliar a análise para as edições mais antigas da PeNSE (2009 e 2012). A escolha do ponto de corte de 60% para as análises de similaridade foi uma opção dos pesquisadores.

Como ponto forte deste estudo, destaca-se a importância da PeNSE como principal instrumento para a vigilância e monitoramento da saúde dos escolares brasileiros. A análise atual visa apoiar os gestores na busca da comparabilidade internacional, identificando a similaridade entre as pesquisas (PeNSE e GSHS). Além disso, a extensa revisão dos questionários permite a identificação das principais mudanças que ocorrem ao longo da pesquisa, facilitando as futuras análises.

Ao longo das edições, a PeNSE apresentou aumento de escolas e escolares participantes, graus de escolaridade, faixa etária, possibilidades de estratificação geográfica dos dados e do número total de questões. Entre as edições de 2015 e 2019, a maioria dos módulos é parcialmente comparável entre si, sendo apenas um totalmente comparável. Em relação à comparabilidade ao GSHS, somente a edição de 2015 foi considerada comparável.

A PeNSE passou por diversas mudanças, tanto nos aspectos metodológicos, como amostra, representatividade e estratificação geográfica, quanto no conteúdo dos questionários do aluno, colocando em risco a estabilidade da pesquisa, além de comprometer a comparabilidade, tanto entre suas próprias edições como com inquéritos realizados em outros países como o GSHS. Tais mudanças podem impedir ações importantes como a análise de tendências temporais dos indicadores de saúde da população adolescente brasileira. Recomenda-se o fortalecimento da comparabilidade da pesquisa brasileira com pesquisas internacionais, e para isso o questionário do aluno da PeNSE deve ser o mais similar possível ao GSHS.

A PeNSE possui notória representatividade e consolidada importância para a produção científica nacional, porém os pesquisadores precisam ter cautela na utilização de seus dados. Análises que envolvem edições diferentes da pesquisa ou dados internacionais por meio do GSHS devem ser cuidadosamente examinadas, a fim de garantir não somente a uniformidade do modo de obtenção de informações, mas também o público-alvo que se busca estudar e sua representatividade.
